# A species-level multi-trophic metaweb for Switzerland

**DOI:** 10.1038/s41597-025-05487-7

**Published:** 2025-07-09

**Authors:** Merin Reji Chacko, Camille Albouy, Florian Altermatt, Joan Casanelles-Abella, Martin Brändle, Victor Boussange, Fadri Campell, Willem N. Ellis, Fabian Fopp, Martin M. Gossner, Hsi-Cheng Ho, Alain Joss, Pascal Kipf, Felix Neff, Andjeljko Petrović, Vincent Prié, Željko Tomanović, Nik Zimmerli, Loïc Pellissier

**Affiliations:** 1 Land Change Science, Swiss Federal Research Institute WSL, Birmensdorf, Switzerland; 2 Institute of Terrestrial Ecosystems, Department of Environmental Systems Science, ETH Zürich, Zürich, Switzerland; 3 Eawag, Swiss Federal Institute of Aquatic Science and Technology, Department of Aquatic Ecology, Dübendorf, Switzerland; 4 Department of Evolutionary Biology and Environmental Studies, University of Zurich, Zürich, Switzerland; 5 Biodiversity and Conservation Biology, Swiss Federal Research Institute WSL, Birmensdorf, Switzerland; 6 Urban Productive Ecosystems, TUM School of Life Sciences, TUM, Freising, Germany; 7 Department of Animal Ecology, Faculty of Biology, Philipps-Universität Marburg, Marburg, Germany; 8 Independent Researcher, Amsterdam, The Netherlands; 9 Forest Entomology, Swiss Federal Research Institute WSL, Birmensdorf, Switzerland; 10 Institute of Ecology and Evolutionary Biology, National Taiwan University, Taipei, Taiwan; 11 Agroecology and Environment, Agroscope, Zurich, Switzerland; 12 Faculty of Biology, Institute of Zoology, University of Belgrade, Studentski trg 16, 11000 Belgrade, Serbia; 13 SPYGEN, 73375 Le Bourget-du-Lac, France; 14 Research Associate - Institut Systématique Evolution Biodiversité (ISYEB), Muséum national d’Histoire naturelle, CNRS, Sorbonne Université, EPHE, Université des Antilles, Paris, France; 15 Serbian Academy of Sciences and Arts, Knez Mihailova 35, 11000 Belgrade, Serbia

**Keywords:** Biodiversity, Ecological networks, Biogeography

## Abstract

Understanding how species interact within ecological networks is essential for predicting the consequences of environmental change, from trophic cascades to broader changes in species distributions and ecosystem functioning across large spatial scales. To facilitate such explorations, we constructed trophiCH: a country-level trophic meta-food web (henceforth “metaweb”) that includes vertebrates, invertebrates, and vascular plants within Switzerland, based on literature published between 1862 and 2023. Our comprehensive dataset catalogues 1,112,073 trophic interactions involving 23,151 species and 125 feeding guilds (e.g., fungivores). Thirty percent of species-level interactions were empirically documented. Additional species-level interactions were inferred by resolving coarser taxonomic records (e.g., inferring links from “species A feeds on genus B”) based on habitat co-occurrences. While explorations of large-scale food webs have often relied on modelling approaches due to data gaps, this empirically based metaweb paves the way for data-driven studies of real-world food webs across space and time. By integrating the metaweb with local species assemblages knowledge, future studies can gain insights into broad patterns of food web structure across spatial scales.

## Background & Summary

Species responses to perturbations have often led to a modification of their geographical distribution^1^ and abundance^2^. Moreover, species are interacting with each other through complex ecological networks in multi-species systems^3^ and are thus additionally exposed to biotic filters that determine the overall response of an ecosystem to perturbations. Consequently, to study the distribution of biodiversity in space and time and flux-associated ecosystem functions (e.g., pollination, herbivore regulation)^4^, a food web approach is a powerful way to describe complex biological communities, taking into account species richness, composition and the fluxes of biomass and energy between them^5^. Yet, the diversity of these biological communities, their associated ecosystem functions, and the efforts to maintain them operate at different spatial (local, regional and global)^6–9^ and temporal^10–15^ scales.

Recent work has demonstrated that the structure and function of food webs vary across environmental gradients at large spatial scales, e.g. along latitudinal, climatic and resource availability gradients^16–21^. Additionally, the dynamics of network structure in mutualistic plant-animal interactions has been demonstrated to vary depending on the temporal scale^22^. Nevertheless, our understanding of how and why ecological networks vary in space and time remains in its infancy, partially due to a shortage of existing interaction datasets and the challenges of comparing differently built food webs^16^. One of the primary hurdles in expanding our understanding of food webs beyond the local scale is the inherent difficulty in collecting empirical data on trophic interactions. The collection of occurrence data alone is costly, time-consuming, and requires taxonomic expertise. The added complexity of observing species in their natural habitats and waiting for trophic interactions to occur—possibly across multiple seasons and life stages—complicates the challenge. Designing and implementing a standardised procedure across habitats and regions to document spatial and temporal variability is unrealistic, given the prohibitive requirements for effort and financial costs. This underscores the necessity for innovative methodologies in the study of ecological networks beyond the local scale.

A meta-food web (henceforth “metaweb”) aggregates all potential trophic interactions between all species that co-occur within a region^23–25^. Local food webs inferred from the metaweb can be considered subsets, similar to how local communities are assembled from a regional species pool^24^. The metaweb approach presents an efficient tool to standardise the comparison of food webs across spatial and temporal scales, and exponential growth in computational power and data collection has popularised the approach in the last five years^26^. Metawebs represent a major step towards understanding complex food web patterns that go beyond the local and the contemporary context^26^. Yet, this larger scale returns the ecologist to the original problem: data gaps are much more evident when regional pools include potentially thousands of species.

In the face of these large gaps, predictive models based on phylogenetic^27^ or morphological^28^ traits may provide an alternative approach. It has long been demonstrated that models based on simple parameters can build complex food webs which are comparable to empirical food webs^29^. For instance, in aquatic systems, body size can be a strong predictor of feeding interactions, and this relationship has been exploited to create the global marine fish metaweb^28^. While such models are useful tools to simplify complex ecological systems, they must be calibrated against empirical data^26^, which can be difficult for already data-deficient regions, taxonomic groups, or interaction types. Additionally, they may not fully capture the complexity, idiosyncrasies, and emergent properties of real-world ecosystems^30^, presenting a need for an empirically based understanding of food webs across space and time.

Empirical metawebs have been constructed across large scales in Europe^20,24,31–33^ and elsewhere^34,35^, demonstrating spatial variations in network structure across elevational^20^, climatic^36^ and anthropogenic^31^ gradients. These metawebs have been generally limited to bitrophic networks (those including two trophic levels of species, such as plants and their pollinators, or parasitic wasps and their hosts) or well-studied guilds, such as tetrapods, which only account for a small fraction of trophic links in the web of life. Plant-animal trophic interactions are one of the primary ways taxa are interconnected in ecosystems^37^, but remain unaccounted for in large-scale studies in Europe—except for some specific taxonomic groups^31,38^. A metaweb that connects the multiple taxonomic groups co-existing in a region by their trophic interactions enables us to integrate species-habitat dependencies with the additional species-species dependencies. Building such a metaweb requires extensive knowledge of species occurrences and their interactions in a defined geographic region.

Historical records of species occurrences are extensive and well-archived for Switzerland, a small country located in central Europe with an area of approximately 41,000 km^2^. Of the nearly 86,000 multicellular species estimated to occur within the country, around 56,000 have been identified^39^, with the spatial distribution of around 10,000 of these species being well-documented^40^. Combining these observations with a method for inferring interaction networks based on geography^41^ can allow for the compilation of a comprehensive food web for Switzerland. This approach has already been implemented for some guilds (birds, orthopterans, lepidopterans and plants)^20^ by making the following assumption: if two species have been observed to interact elsewhere, the interaction may also be realised if they co-occur within a spatially confined unit. The assumption fixes the diet breadth of a species across the entire metaweb without accounting for intraspecific diet variation driven by biotic^42–46^ and abiotic^47,48^ factors. We emphasise that the nature of the metaweb approach creates a network of all potential links between the target species. This is, in fact, an overestimation of any species’ diet breadth at any one point in space and time. We refine this inference approach^41^ by only including potential links for documented interactions with known co-occurrence within the region (Switzerland) and by further trimming inferred interactions based on species’ habitat associations. Additionally, local food web structure has been demonstrated to be influenced more by the assembly process than local dynamical processes^49^. Thus, by restraining interactions by species ecology (habitat associations), distribution, and the assembly process, local food webs inferred from the trophiCH database remain within a “realistic boundary” of potential interactions^20^ while forming comparable local food webs built from the same metaweb.

**Fig. 1:**
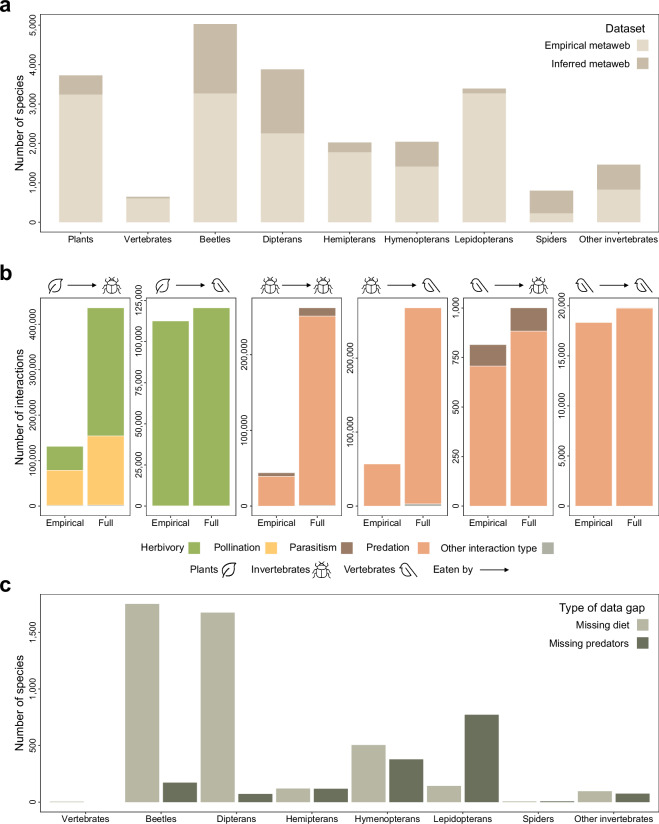
Distribution of species, interactions and data gaps within the metaweb. (**a**) The distribution of taxa covered by the empirical and full metawebs, (**b**) the distribution of interactions in the empirical and full metaweb, separated by interactions between plants, invertebrates and vertebrates, and interaction types (herbivory, pollination, parasitism, predation and other interactions missing information on interaction types). (**c**) the distribution of data gaps in the metaweb by broad taxonomic groups, separated by species missing diets and species missing predators. Icon attribution: Flaticon.com.

Here, we provide the trophiCH dataset, an empirically based species-level metaweb for 26,243 taxa (including vertebrates, invertebrates, and vascular plants) in Switzerland and 1,188,063 links between them. Of these, 1,107,253 interactions between 23,002 taxa are resolved to the species–species level (Fig. 1a,b). This metaweb is based on data extracted from 732 sources of scientific and grey literature (published papers, books, voluntary websites, etc.). For some taxa, we additionally used a spatial model based on simple parameters (co-occurrence in the same habitats and vertical strata) to infer trophic information from the genus and family levels to the species level. We provide the species list used in this study along with information on the associations of these species with habitats and their vertical strata in those habitats. We provide a dataset including reference metadata, such as the full citation, publication date, location, accession, and data collection methods for each reference.

Our multi-taxa metaweb is an archive of potential interactions that may occur between species, if they co-occur within the confines of our region: Switzerland. When combined with high-resolution occurrence data, it can infer more localised networks, which can be used to facilitate comprehensive large-scale explorations across Switzerland^40^. As such, structural and topological properties of food webs (such as connectance^50^, modularity^51^, nestedness^52^, etc.) can be compared across environmental gradients (such as precipitation, land-use intensity, temperature, etc.)^16,25^. For example, the trophiCH metaweb was combined with existing classifications of species’ associations with biogeographic regions to predict twelve regional multi-habitat biogeographic food webs^53^. By additionally utilising the habitat-associations presented in this present data descriptor, the robustness of these twelve food webs to different types of sequential species extinctions due to habitat loss were assessed, demonstrating elevational differences^53^. In another example, aquatic and terrestrial communities were first sampled using environmental DNA along an urbanisation gradient in the city of Zurich, Switzerland. By combining the trophiCH metaweb with the sampling results, 54 local site-level food webs were inferred, and used to demonstrate that combined aquatic and terrestrial food webs become decoupled and more homogeneous along an urbanisation gradient^54^. In yet another case, the trophiCH metaweb was combined with species distribution models (SDMs) to build nearly 18,000 catchment-scale food webs across Switzerland^55^, each representing a spatial resolution of ca. 2 km^2^. Local species assemblages for river sub-catchments were first inferred using SDM-based catchment-scale presence-absences, and food webs were built by inferring that species co-occurring within the catchments and sharing trophic interactions within the trophiCH metaweb would inherit the interactions at the catchment scale^55^. This study not only demonstrated that catchment-level food web properties were shaped by land use and elevation, but also combined SDM outputs and trophic interaction data to develop a metric of habitat connectivity accounting for species’ resource availability^55^. Other such potential large-scale, high-resolution studies of environmental gradients are especially relevant in the face of a recent publication of a 25-m resolution multi-raster dataset at the country-scale for Switzerland, covering environmental variables across topographic, bioclimatic, edaphic, land use and vegetation categories, among others^56^.

While most existing metawebs have only been used to study spatial variation^20,25,28,31,32,34,47,57–60^ (with some exceptions^21,33^), by incorporating temporally explicit data as such available in Switzerland^61^, it may be possible to study the temporal dynamics of these local networks. In the face of gaps in data needed to infer local networks, the metaweb itself can still provide crucial information on the topography of the regional food web. For example, robustness analyses simulating real-world perturbations (such as the loss of species or of interactions)^18,62^ on threatened habitats across the multi-habitat metaweb could provide an understanding of how habitat-specific losses could influence food web structure and stability. Utilising the entire metaweb could enable us to also consider variations in dietary preferences within species to identify the potential of rewiring the food web, which could lead to new interactions that may only occur in the future as species distributions shift. Metawebs have also been used to predict not only local networks, but also entire metawebs in similar biomes^63^, and the Swiss metaweb may be a first step towards a multi-trophic food web for other European countries, or even the whole of Europe. Finally, the metaweb can be used for Swiss-scale conservation measures. For example, graph-theoretic topological metrics, such as betweenness or closeness centrality^64^, can be used to identify key species for conservation.

## Methods

### Checklist of species

We compiled a checklist of animal species based on existing literature for Arachnida^40,65,66^, Diplopoda^67^, Entognatha (Diplura^68^). Insecta (Coleoptera^69–88^, Dermaptera^68^, Diptera^89,90^, Ephemeroptera^91^, Hemiptera^92–95^, Hymenoptera^68,96–105^, Lepidoptera^40,106^, Mecoptera^68^, Megaloptera^107^, Mantodea^107^, Neuroptera^68^, Odonata^108^, Orthoptera^109^, Plecoptera^91^, Raphidioptera^68^, Strepsiptera^68^, Thysanoptera^68^, Trichoptera^91^ and Zygentoma^110^), Crustacea (Amphipoda^111–115^ and Decapoda^116^), Mollusca^117^ and Vertebrata (Hyperoartia^118^, Actinopterygii^118^, Amphibia^40^, Aves^119^, Mammalia^120^, Reptilia^40^). For plants, we used the Tracheophyta^121^ checklist of Switzerland. We predominantly used federal checklists, supplemented by continental checklists where data were lacking, as follows. For a checklist of Swiss aphid parasitoids, we selected a subset of the checklist of the *Aphidiinae of the Czech Republic*^104^, which was validated by a local expert (see: Acknowledgements). The existing Chrysididae^103^ checklist was similarly validated, as significant changes had been made since the publication of the previous list in 1997. In total, this checklist consisted of 24,039 species. We emphasise that this checklist is not meant to be used as a comprehensive checklist for each family presented here but includes all the species for which trophic and/or occurrence information was available. For example, for some families, such as Chrysomelidae (Coleoptera), we only include an incomplete set of species known to occur in Switzerland (334, in comparison to 399 species according to GBIF records^122^, or potentially 86%). Our aim was to include as many well-documented species as possible, especially for groups, such as Chrysomelidae, where validated checklists may be missing, but trophic information is readily available.

### Literature-based data search and extraction

We systematically searched for primary literature and datasets using the Google Scholar^123^ and Google Dataset Search^124^ engines, respectively, and for books using swisscovery, the Swiss platform for sharing scientific information between around 500 libraries^125^. We used every combination of the following search queries: taxonomic names at the order, family, and genus level (for animals), the ecological terms “trophic”, “diet”, “prey”, “predator”, “host”, and “interaction”, and the spatial terms “Switzerland”, “France”, “Germany”, “Austria”, “Italy” and “Europe” (the regions surrounding Switzerland). We included 305 unique sources from books^66,94,96–104,108,109,111,116,118,120,126–262^, primary literature^18,24,25,92,263–399^, and existing databases^400–409^. Pairwise species interactions between the resource and consumer taxa were additionally filtered to include only taxa present in our Swiss checklist.

For larger databases, specific approaches were needed for further extraction. For birds, we directly consulted the institutional website of the Swiss Ornithological Institute^410^ by searching species names and manually extracting diet information from the “food” section of each species page. For the GloBI database, we downloaded stable version 0.3^411^. We included only the rows in the taxonomic names in the columns “sourceTaxonName” and “targetTaxonName” which matched the taxonomic names in our checklist of taxa. We additionally only included interactions from the “interactionTypeName” columns which matched the following terms: “visitsFlowersOf”, “parasiteOf”, “parasitoidOf”, “eats”, “visits”, “pollinators”, “hasHost”, “mutualistOf”, “preysOn”, “ectoparasiteOf”, “kleptoparasiteOf”, and “endoparasiteOf”. For the Animal Diversity Web^405^, we used the associated Quaardvark tool^412^ to extract the data. With regards to the query “What groups of animals are you interested in searching?”, we chose for the “Animal Group” selection the kingdom “Animalia” and for the “Geographic Range > Biogeographic Regions” selection the term “Palearctic”. For the report on “What do you want to know about them”, we choose the taxonomic rank of “Species”. In the “Habitat” selection, the following terms were included: “Terrestrial Biomes”, “Aquatic Biomes”, “Wetlands”, and “Other Habitat Features”. For the “Food Habits” selection, the following terms were included: “Primary Diet”, “Animal Foods”, “Plant Foods”, “Other Foods”, “Foraging Behavior”. Additionally, from the “Predation” selection, we chose the “Known Predators” option. From the resulting dataset, we excluded all interactions with taxonomic terms which were not present in our checklist. For the “freshwaterecology.info” database^403^, we searched under the “Macro-invertebrates” sub-section as follows: First, we selected all taxa listed in the “Taxagroup” section and searched for all information on the ecological parameter “feeding type”^413^, based on the Moog (1995) classification^414^. Of the ten terms, the term “other feeding type” was discarded. For “grazers/scrapers” we translated this to include the feeding guilds “Algae”, “Detritus” and “POM” (particulate organic matter), “miners” were translated to include “Algae” and “Plantae”, “xylophagous taxa” (feeding on woody debris) were translated to the term “Detritus”, “shredders” were translated to include “Plantae”, “POM” and “Detritus”, “gatherers/collectors” were translated to “POM”, “active” and “passive” filter feeders were translated to “POM” and “Microprey”, while “predators” and “parasites” were translated to “Animalia”, while the Interaction_Type column specified whether this referred to “Predation” or “Parasitism”.

Following an initial validation of data completeness (see: Technical Validation), we conducted a secondary search for additional interaction information, focusing on species with no data identified in the preliminary search. This involved targeted Google searches using the species name in combination with the following terms: “diet,” “food,” “prey,” and “host”, and limiting our search to the first search page. These searches led us to 53 additional sources, including naturalist websites, species fact sheets, and voluntary science platforms.

For many invertebrates, we used BugGuide^415^, by searching the missing species names and manually extracting diet information from the “Diet” section of each species page. For other taxa, additional sources were accessed directly through the targeted Google searches, including websites from Wikipedia^416^, the Woodland Trust^417^, the National Wildlife Federation^418^, the Australian Faunal Directory^419^, the Aquatic Insects Key^420^, Natura Bohemica^421^, Lepidoptera and other life forms^422^, Pyrgus^423^, the UK leaf and stem mines of British flies and related insects^424^, Animalia^425^, Encyclopedia of Life^426^, Artsdatabanken^427^, the Plecoptera Species File^428^, the UK Beetle Recording^429^, the Penn State Agronomy Guide^430^, the University of California Case Histories Biological Control Project^431^, Project Hypersoil^432^, Influential Points: Statistics and Aphids, things that bite and suck^433^, Lepidoptera Mundi^434^, LepiWiki^435^, the Moths and Butterflies of Europe and North Africa^436^, info fauna^437^, Microlepidoptera: Atlas van de kleine vlinders in Nederland^438^, Association Papillons de France^439^, The Reptile Database^440^, Soil Ecology Wiki^441^, ThripsWiki^442^, Trichoptera Ireland: the distribution and autecology of caddisflies (Trichoptera) in Ireland^443^, UK Beetles^444^, Wiki der Arachnologischen Gesellschaft^445^, the Online Database of Afrotropical Moth Species^446^, Bee-Finder^447^, British Bugs: an online identification guide to UK Hemiptera^448^, the Bees, Wasps & Ants Recording Society^449^, Chrysis.net^450^, Butterflies & Moths of Palaearctic Regions^451^, Beetle Fauna of Germany^452^, Lepi’Net: Les Carnets du Lépidoptériste Français^453^, The Atlas of Common Freshwater Macroinvertebrates of Eastern North America^454^, Heuschrecken-Wiki^455^, Schmetterlinge der Schweiz - Butterflies & Moths of Switzerland^456^, The Sawflies (Symphyta) of Britain and Ireland^457^, Spektrum^458^, Thrips-iD^459^, UKmoths^460^, Wildbienen^461^, Faszination Wildbienen^462^, Insektenbox^463^, Life in Freshwater^464^, Meadowia^465^, and Naturspaziergang^466^. As individual species pages and content authors varied across these platforms, we recorded the specific URL for each source page directly within the references meta-dataset (see: Data Records). Each interaction derived from one of these websites is therefore linked to its exact source page in the dataset, even though only the main website URLs are cited in this data descriptor. Data collection, extraction and archival occurred between January 2021 and October 2023. The temporal range of the covered resources were from 1862–2023.

We extracted digital data, when possible, using an automated pipeline in R^467^ (version 4.3.2) and RStudio^468^ (version 2023.12.1) and saved them as comma-separated files. Books and other analogue data were extracted through manual input into comma-separated files. We primarily recorded resource and consumer names and their taxonomic ranks, then we translated German and English names into scientific names, where applicable. Where available, we gathered additional information on associations to a species’ habitat and to a position in the vertical stratification of the ecosystem, on their life stages, and interaction type. Broad non-taxonomic diet information (e.g., detritivory) and diet information on non-focal taxa (e.g., fungi) were recorded and standardised into 125 specific feeding guilds. These also included some arthropod families (mostly ill-studied dipterans and beetles) for which higher-resolution data were unavailable. All datasets were merged into one large comma-separated file (henceforth raw dataset)^469^. The raw dataset consisted of 442,599 interactions between 22,862 taxa, based on 655 unique references. Of these, 364,136 interactions were documented between 16,907 species (Fig. 1a,b). All detailed references for the interactions are provided as a meta-dataset.

### Taxonomy-based inference of interactions

**Fig. 2:**
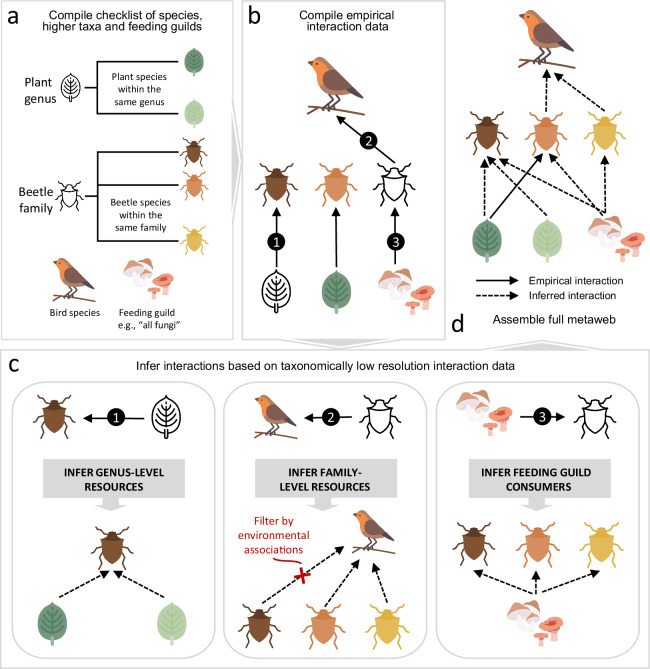
Construction and expansion of the metaweb. (**a**) Initial compilation of a species checklist, as well as their upstream taxonomic information for genera and families, and creation of feeding guilds, such as fungi. (**b**) Empirical data collection process, focusing on information at the species, genus and family level. (**c**) We expanded links where species were known to consume an organism at the genus level to include all species within the genus (link 1). We additionally inferred links where species were known to consume an organism at the family level, for generalist predators, and filtered by their associations to habitat and vertical stratum within the environment (link 2). Links were also inferred where it was explicitly known that a family of organisms were generalist feeders on feeding guilds, such as fungi (link 3). (**d**) A final metaweb is assembled using all empirical and inferred links. Icon attribution: Flaticon.com.

While the raw dataset included many species-species interactions, many other interactions were recorded with the consumer taxa at higher taxonomic levels. However, it has been demonstrated that varying node resolution within observational ecological networks can modify network topology metrics^470^. Additionally, our raw dataset included many hierarchically nested interactions, such as genus A and species B eating species C, wherein species B is within the genus A, creating artificial interaction redundancies. Thus, to retain metric reliability in future analyses of the metaweb without losing potential trophic data (as some species only had low-resolution resource information) and to reduce some redundancy of interactions, we implemented further strategies to increase the taxonomic resolution of the metaweb (Fig. 2, see below).

We initially followed the approach used by Maiorano *et al*.^24^: if a species was described as consuming species from the family level or higher, all species within the described families were considered as potential prey. While a valid approach for tetrapods, this approach can be problematic when considering interactions between insects and plants. Phenotypic variation within families of insects, which at times include hundreds, if not thousands of species, is higher than within tetrapod families. Thus, we restricted such taxonomy-based inferences to the genus level (Fig. 2, link 1). Some family-level inferences were allowed for pollinators, but only if empirical records explicitly confirmed the species to be polylectic. The remaining genus-level information was retained in the metaweb at the lower taxonomic resolution.

The species-level trophic interactions within the raw dataset contained a strong bias towards well-studied specialist taxa, especially for primary consumers such as lepidopteran larvae. In contrast, the diets of generalist consumers such as spiders were often only classified to the family level or higher. To better account for the generalist predators in the metaweb, we implemented an empirically based interaction inference strategy based on co-occurrence in the habitat and vertical stratum within ecosystems (see: Habitat and stratum associations). Firstly, all families within the checklist containing only generalist predator species were identified. For species within these families, documented information on their prey at the family level was first used to infer interactions from the predator species to all species within the family. Subsequently, we removed interactions in which the resource and consumer species do not potentially share habitats and vertical strata within the habitats (see: Habitat and stratum-associations and Fig. 2, link 2).

In cases where families have been documented to be generalised consumers of taxonomic groups that are absent from the metaweb but present in the form of feeding guilds, we inferred links between all species within the family and the feeding guilds. For example, all larvae of the Platypezidae fly family feed on fungi^403^. Since we grouped all fungi into the basal feeding guilds “Fungi” within the metaweb, all Platypezidae species known to occur within Switzerland were thus connected to the node “Fungi” (Fig. 2, link 3). In this way, information on the predators of Platypezidae species did not have to be aggregated to the family level.

### Habitat-association and position in the vertical stratification of the habitat

We define the habitat associations of each species according to nine classes in the broadest of the TypoCH^471^ habitat classifications. The habitat information was collected in two different ways. Firstly, we collected it along with interaction data where available. Secondly, we inferred habitat associations by intersecting species occurrence data^40^ with the Habitat Map of Switzerland^472^. We used the *st_intersection()* function from the *sf* package^473,474^ (version 1.0–15) to intersect the point data with the polygonal habitat map. The output provided occurrence counts per species and habitat. We retained all habitat associations with at least 100 counts, as well as associations that had also been documented in the literature survey. Then, we classified all species with three or more habitat associations as habitat generalists and all species with less than three as habitat specialists. We justify this as species with two habitat associations may still be specialists, where different life stages may have high habitat specificity. For habitat generalists, only habitat associations with occurrences above the median of total occurrences were retained, others were dropped. For habitat specialists, habitat associations were only retained if the species was documented at least five times within that habitat. We obtained 6,818 habitat associations based on the Habitat Map and 3,062 based on observational data. For the remaining species, we inferred habitat associations. Firstly, for species where habitat associations were missing, we first combined all known habitat associations of all species within the same genus. We only retained the habitats shared by the median number of species within the genus or higher and assigned these to the species where habitat data were missing. Thus, we were able to infer habitat associations for all species; 18% of plant species habitat associations were inferred at the genus level. For animals, this genus-level inference created habitat associations for 16% of all animal species. A similar inference was made for another 51% of the animal species habitat associations, but using aggregated family-level habitat associations, as genus-level inferences were not possible due to gaps in data.

We defined the incidence of each species according to the following strata within a habitat: on ground or in leaf litter, on vegetation, in ground, in water, on host, in dwellings, in air, in host nest, in vegetation, on fungi, in caves. While most of these classes refer to the position in the vertical stratification, some, such as “in vegetation” were included to separate free-living species which can feed on multiple organisms, from species which living within a plant, and thus would not be available as a potential resource for an organism only feeding on the outside of the vegetation. This information was collected along with interaction data for 10,360 animal species (as well as for relevant genera and families if species-level information was not available). All 3,775 plant species were classified according to the Raunkiær plant life-form classifications in *Flora Indicativa*^475^. Plants classified as hydrophytes or pleustophytes were classified as “in water”, epiphytes were classified as “on vegetation”, and all others were classified as “on ground or in leaf litter”, “in ground” and “on vegetation”, the latter such that inferences could be correctly made between animals classified as “on vegetation” and vegetation. Where information was unavailable, species-level characteristics were inferred using the same methods as for the habitats, first at the genus level (3,886 species) and then at the family level (6,119 species). Thus, 49% of animal associations to vertical strata in their habitats were inferred from family or genus-level information.

## Data Records

We provide all data and scripts^469^ on Envidat, the Swiss data portal for environmental monitoring and research data. We provide six datasets: 1) the metaweb, 2) the taxa checklist, 3) the data source meta-dataset, 4) the list of generalist basal and predator families and polylectic species with citations and 5) a dataset with citations for the inferences of missing predators and 6) a dataset with citations for the parallel inference of diets from similar species (see: Data completeness). The metaweb is a pairwise interaction dataset, with each row representing a potential interaction (see Table 1 for all column information). This dataset includes the taxonomic names, ranks and life stages (where available) of each species in the interaction. Moreover, we provide a numerical identification (Citation) column for the citation, which relates to the full citation information provided in the resource metadataset. We additionally include information on the level of inference by taxonomic expansion (see: Taxonomy-based inference of interactions), as well as information on further details on the type of interaction, such as predation, parasitism, or pollination, where available. The taxa checklist provides our list of species and feeding guilds, upstream taxonomic information, and their associations with habitats and vertical strata within habitats (Table 2). For each citation number, the resource meta-dataset contains a full APA-style citation, information about the data source and the methods used to collect the datasets, a stable accession, as well spatial and temporal information about the data collection (Table 3). Additionally, we include a dataset listing the taxa for which the diet breadth was broad, for families of predators and those using feeding guilds as resources, and for polylectic species, along with a citation ID for the relevant citations (Table 4). We provide a dataset listing the families for which predators were missing, and inferred based on broad data, with accompanying citations (the structure of this dataset is identical to Table 1, except it is missing the column named “Inference”). Lastly, we provide a list of species for which diet information was inferred based on taxonomically similar species, with references validating their similarities in diet (Table 5).

**Table 1:** The data structure of the metaweb dataset.

Column name	Description	Example(s)
Source_Name	The name of the source taxon, i.e., the consumer	*Perca fluviatilis*
Target_Name	The name of the target taxon, i.e., the resource	*Heptagenia sulphurea*
Source_Rank	The taxonomic rank of the source taxon	Species
Target_Rank	The taxonomic rank of the target taxon	Species
Source_Life_Stage	The life stage of the source taxon, if available and/or application. The stages have been summarised into “Young” or Adult”. For insects, “Adult” refers to the imaginal stage, while all larval stages have been summarised into the “Young” stage.	Young and Adult, Young, Adult
Target_Life_Stage	The life stage of the source taxon, if available and/or applicable. The stages have been summarised into “Egg”, “Young” or Adult”. For insects, “Adult” refers to the imaginal stage, while all larval stages have been summarised into the “Young” stage.	Egg, Egg and Young, Young and Adult, Egg, Young and Adult, Adult, Young, etc.
Citation	The ID number(s) of the data source(s) documenting the interaction between the source and target taxa. These values correspond with the Citation column in the citation meta-dataset.	261, 192, etc.
Inference	Purely empirical interactions are marked NA in this column. For other inferred interactions, this column specifies the degree to which the interaction was inferred. The categories and their explanations are further expanded within the dataset’s metadata.	Source_Species_Target_Family, Source_Species_Target_Genus
Interaction_Type	A more detailed classification of the type of interaction.	Predation, pollination, herbivory, etc.
ID	A combination of the Source_Name and Target_Name columns to provide the final interaction ID	*Perca fluviatilis - Heptagenia sulphurea*
ID_og	A combination of the Source_Name and Target_Name according to the original empirical interaction ID. For empirical interactions, this is identical to the ID column.	*Perca fluviatilis - Heptagenia*

**Table 2:** The data structure of the taxa checklist.

Column name	Description	Example
Taxon	The name of the taxonomic unit	*Dysaphis apiifolia*
Rank	The taxonomic rank of the taxon	Species
Kingdom	The taxonomic kingdom within which the taxon resides	Animalia
Phylum	The taxonomic phylum within which the taxon resides	Arthropoda
Class	The taxonomic class within which the taxon resides	Insecta
Order	The taxonomic order within which the taxon resides	Hemiptera
Family	The taxonomic family within which the taxon resides	Aphididae
Genus	The taxonomic genus within which the taxon resides	*Dysaphis*
Species	The species epithet of the taxon if it is resolved at the species level	*apiifolia*
Habitat	The habitat association (s) of the taxon	Grassland, Forest, etc.
Zone	The associations of the taxon to the vertical stratum (or strata) in the habitat	On vegetation, In water, etc
Count	A total count of the number of occurrences documented in Switzerland per taxon, where available.	156, NA
Hab_Citation	The ID number(s) of the data source(s) documenting the habitat-association(s) of the taxon. These values correspond with the Citation column in the citation meta-dataset.	450, 390, etc
Hab_Inference	Purely empirical associations are marked NA in this column. For other inferred associations, this column specifies the degree to which the information was inferred.	Family, Genus
Zone_Citation	The ID number(s) of the data source(s) documenting the association(s) of the taxon to the vertical stratum (or strata) in the habitat. These values correspond with the Citation column in the citation meta-dataset. In cases where the associations were inferred by pooling all species within the same family or genus, the cell is marked NA.	450, NA
Zone_Inference	Purely empirical associations are marked NA in this column. For other inferred associations, this column specifies the degree to which the information was inferred.	Family, Genus

**Table 3:** The data structure of the reference meta-dataset.

Column name	Description	Example(s)
Citation	The ID number(s) of the data sources(s) documenting the interaction between the source and target taxa. These values correspond with the ID column in the metaweb interactions dataset.	18
Full citation	APA-style full citation of the data source	Benadi, Hovestadt, T., Poethke, H.-J., & Blüthgen, N. (2014). Data from: Specialization and phenological synchrony of plant–pollinator interactions along an altitudinal gradient [dataset]. Dryad. 10.5061/dryad.8mn44
Resource type	Classification of the resource into broad types.	Primary literature, voluntary science, expert opinion, etc.
Resource type comment	Additional comments on the classification of the resources into	This text primarily focuses on species identification, with some information on their biotic interactions.
Methods	When available, classification of the data collection methods into broad types	Molecular methods, morphological analysis, visual observations, etc.
Methods comment	For some method classifications, a more detailed summary of the work	DNA metabarcoding of gut content, DNA metabarcoding of pollen, etc.
Location	The spatial range at which this information was collected	Europe
Year	The publication date of the data source. This was used instead of the data collection date, as the collection dates for larger archived datasets were often unavailable.	1995
Accession	A form of stable accession for the data source	978-32-5807-461-0, 10.5519/HAVT50XW, etc
Accession type	The type of stable accession that is provided.	ISBN-13, DOI, URL, etc
Data Type	This category refers to the way the data was extracted, digital for datasets extracted automatically through R scripts and analogue for text or books that required handling by a human.	Digital, Analogue

Please note that the examples do not all arise from the same Citation ID.

**Table 4:** The data structure of the diet range dataset.

Column name	Description	Example(s)
Taxon	The taxonomic name of the potential consumer	Accipitridae
Range	The diet range of the taxon	Basal (if they are generalists feeding on a feeding guild, such as a family of detritivorous insects), Predator, Polylectic (for polylectic pollinators, only if they have explicitly been classified as such in the literature)
Citation	The ID number of the reference(s) documenting this information about their diet and potential diet range	301, 491
Rank	The taxonomic rank of the taxon	Family, Species

Please note that the examples do not all arise from the same Citation ID.

**Table 5:** The data structure of the dataset summarising special cases.

Column name	Description	Example(s)
Taxon	The taxonomic name of the consumer or resource species	*Anguis vernonensis*
Inference_Taxon	The taxonomic name of the species from which inferences are made	*Anguis fragilis*
Citation	The ID number of the reference documenting the diet or predator similarities between the two taxa	508
Case	Identification of whether the taxon is missing diet or predator information	Missing diets

Please note that the examples do not all arise from the same Citation ID.

## Technical Validation

### Data collection

**Fig. 3:**
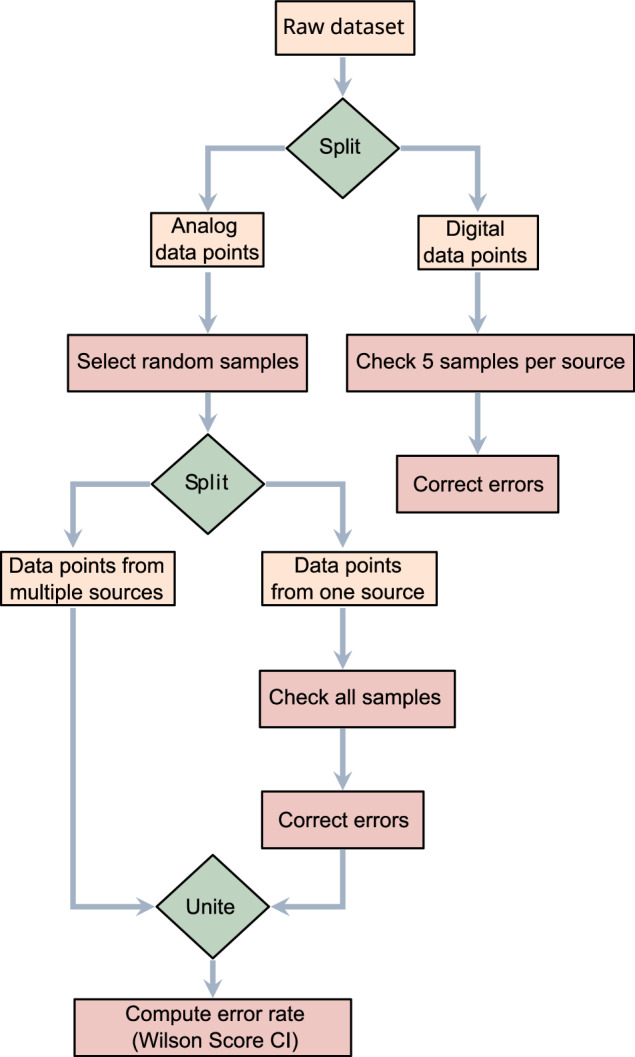
Validation of the data extraction process. The diagram outlines the sequential steps, beginning with the division of raw datasets into analogue and digital data, then random sampling, error checking, and consolidation across multiple data sources, culminating in the Wilson Score Confidence Interval computation.

We aimed to estimate the human error arising from manual and automated data extraction (Fig. 3). We first classified the data as originating from either analogue or digital sources. Analogue sources include data which were manually transcribed into comma separated values. Digital sources include data which were received as data tables or matrices, where the transformation to the standard data table format was automated through scripts in R. Since we processed digital sources automatically, we assumed the error rate to be either very high or close to zero. Thus, five random samples (or the maximal possible number if the data sources included fewer than five samples) were validated for each digital dataset (Fig. 3). One error was discovered due to an error in the script, which was corrected, such that the error rate was refactored to be 0 for the digital sources.

For analog sources, we aimed to estimate a (Wilson score) confidence interval of the error rate. To achieve a 95% confidence level (*z = *1.96), with a margin of error e = 0.01, and an estimated error rate p = 7%, the required size of the random sample was of n = 2501, based on the following approach: $n=\,\frac{{z}^{2}p(1-p)}{{e}^{2}}$. The estimated error rate was based on the validation approach of the European tetrapod metaweb^24^, which estimated a base error rate of 6%. We then randomly sampled 2501 data points and validated them as follows: if the data point had been further confirmed by at least one other data source in the metaweb, it was assumed to be correct (Fig. 3). For all other data points in the sample, we manually checked each source and discovered three errors in total, all of them single-entry errors. The error rate for the analogue data sources was thus computed to be between 0.04% and 0.35% (95% CI). This validation was conducted in Python (v. 3.11.4)^476^ using *numPy* (v. 1.25.1)^477^, and *pandas* (v. 2.0.3)^478^.

### Data completeness

To assess the extent of data gaps, we checked whether trophic chains were truncated by comparing true basal and apex taxa to those appearing as such within the metaweb. True basal taxa were identified as vascular plants and non-animal groups (such as detritus) within the feeding guild classes (3,903 taxa). True apex taxa were classified as apex predators and parasitic arthropods of apex predators (1,018 taxa). We additionally identified taxa for which the only trophic information was a self-loop, in essence, obligate cannibals. The raw network topology revealed that 2,170 species were improperly in an apex predator position (due to missing outgoing links), while another 5,581 species were improperly in a basal position (due to missing incoming links).

We focused on filling these gaps for species for which spatial data are readily available, as many other species for which information is lacking are less well-studied. Moreover, we aimed to cover generally well-studied groups completely, such as tetrapods. To this extent, we conducted broader internet searches to obtain information from secondary and grey literature sources, such as voluntary scientists and insect enthusiasts (see: Literature-based data search and extraction). For seven species (the slow worm, five orthopterans and one caddisfly), predator information was present while diets were missing. Their diets were inferred based on the diets of ecologically similar species, after confirmation in the literature with regards to the similarities in diets. Additionally, for one species of spider, the predators were inferred based on the predators of an ecologically similar species. Many families of arthropods were additionally missing information on predators. In a few cases, such as with wood beetles, we inferred the predators of the species in these families to generalist predator families such as woodpeckers, or inferred predators of diplopods based on broad information such as, “hedgehogs feed on diplopods”, to hedgehog species being connected to all diplopod species within the same shared habitat. Some trichopteran predators were inferred based on the fish predators most commonly shared by other trichopterans. This approach was also applied to amphipods missing predators based on amphipod-eating fishes and birds, as well as decapods based on decapod-eating fishes and birds. In these cases, the interactions were further trimmed by habitat and vertical stratum. Each case is documented by an accompanying citation and classification in the “Inference” column such that this uncertainty can be accounted for in future usage (see also, Code Availability: 02_inferring_interactions_special_cases.R for more detail on all such special cases). After this correction, we recovered diet information for 1,221 species and predator information for 574 species. Nonetheless, 4,298 species do not have any diet information, while 1,594 species have no predator information. These gaps shine a light towards potential future lines of research. We additionally view the identification of these gaps as a starting point for future contributions to the trophiCH database. While the current version of the metaweb is static, we welcome researchers with interaction data—particularly for understudied taxa or ecological groups like beetles and dipterans (Fig. 1c)—to contact the corresponding authors if they are interested in contributing to future updates or extensions of the dataset, or to correct/validate the modelled interactions.

### Comparison to other empirical metawebs

**Fig. 4:**
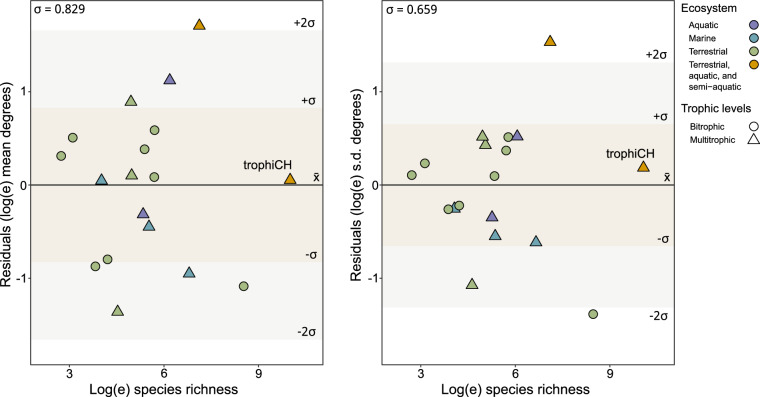
Comparison of trophiCH to other metawebs. The scatterplots compare log species richness to the residual variation from mixed linear effects models predicting mean degree (left) and standard deviation of degree (right), treating the type of network as a random effect. The colours of the data points represent the type of ecosystem (aquatic: purple, marine: blue, terrestrial: green and terrestrial, aquatic, semiaquatic: orange). The shapes represent the type of network (circle: bitrophic, triangle: multi-trophic). The dark beige rectangles represent the first(±σ) and the light beige rectangles represent the second (±2σ) standard deviations from the mean(x̄).

We compiled a list of eighteen existing empirical metawebs^31–33,479–489^ to facilitate a relative comparison to the data coverage of our metaweb (Supplementary Information Table S1). We focus on total degree, which considers the sum of each species’ in and out links in a network. We calculated the mean of the sum of all species’ total degrees to consider the data coverage, and the standard deviation of the mean. These metrics are likely to depend on the spatial range of the network (some metawebs were limited to one city while others were global) and species richness, as well as random or sampling effects, and therefore cannot be robustly compared across networks^16^. To control for these spurious effects, we modelled the co-variation of network properties with linear regression and compared residuals of the metrics^16,50^. We fit linear mixed effects models to predict relative mean total degree and its standard deviation based on relative species richness, treating the type of network (bitrophic or multitrophic) as a random effect. We used the *lmer()* function from the *lme4* package (v. 1.1–35.1)^474,490^ in R. We then compared the mean of residuals to the values of our metaweb to determine whether our residual values were outliers (values outside two standard deviations of the mean). For both metrics, our metaweb remained within two standard deviations (Fig. 4). The European tetrapod metaweb was the only outlier for both metrics. Thus, for its size, this metaweb contained relatively more interactions between the species, and a relatively larger variation in the number of links per species. This result is understandable, as this metaweb has a strong bias towards well-studied organisms (tetrapods)^491^, in a geographic region where biodiversity had historically been relatively better-studied (Europe) than other parts of the globe^492^. Although data gaps remain (see Technical Validation: Data completeness), we argue that our trophic data coverage for species is on par with other existing empirical metawebs. To our knowledge, trophiCH represents the largest empirically based metaweb in existence, both in terms of species richness and trophic levels. In comparison, the next largest metaweb (see Supplementary Information Table S1) contains a fifth of the species richness and only includes plant-frugivore interactions.

## Usage Notes

This work compiles a large empirically based dataset of species interactions along with species-species interactions inferred based on empirical interactions known at lower taxonomic resolution. The temporal span of our sources (1862–2023) reflects the accumulation of ecological knowledge over time. Indeed, over 95% of the interactions documented within our empirical metaweb were published after the year 2000. It should be noted, however, that the publication year does not necessarily correspond to the date of observation, which is often not reported in books. This additionally introduces a methodological bias: older sources often document only a few observed interactions per taxon, while modern approaches—particularly metabarcoding and database aggregations—can yield hundreds or thousands of interactions in a single dataset. As our metaweb documents potential interactions filtered by present-day species occurrence in Switzerland, the original publication year does not imply that the interaction still occurs today, but rather that it is ecologically plausible. We emphasise again that this is a metaweb, including many interactions that may not be realised at any singular point in space and time. Users should be aware of these methodological and temporal heterogeneities when interpreting the data.

Additionally, when existing metawebs were incorporated into our metaweb, some of the data had already been inferred based on empirical knowledge. For example, the bird-plant interactions in the Swiss bird-lepidoptera-plant metaweb^20^ were inferred based on expert knowledge of broad diet preferences and habitat-associations. The metaweb serves as an archive that ecologists can use, for example, to create their own local networks – using local occurrence data or simulations. The complete transparency of our metaweb with regard to the derivation of the individual interactions and their data sources enables customization to the individual needs and requirements of the users. Future studies should check the quality of each data point with regards to their research aims before using the dataset in its complete form. Moreover, we note that our metaweb approach does not provide quantitative information about the importance or abundance of each interaction (i.e. weighted interactions). Hence, the derived food webs provide qualitative and not quantitative insights on ecological networks. Finally, a metaweb approach is, by definition, dependent on the assumption that species that interact in the metaweb will always interact at the local scale if they are found to co-occur^16,23^. This collapses local scale variation due to abiotic and biotic variation, and only accounts for variation in interactions due to shifting species distributions. Future work should aim to incorporate spatial variation in both the occurrence and strength of interactions, which will require new data and methodological advances.

For the rapid visualisation and exploration of the dataset, we additionally host an R Shiny application, available at: https://webapps.wsl.ch/trophiCH.

## Supplementary information

Supplementary Information 1

## Data Availability

We provide five scripts, accompanying functions, and the raw data required to run these scripts to reproduce the taxonomic expansion and validation of the datasets^469^. In the first script (01_inferring_interactions.R), we infer interactions using genus and family level interactions and for basal feeding guilds (see Methods: Taxonomic expansion). In the second script (02_inferring_interactions_special_cases.R), we infer further interactions for a few special cases with detailed explanations. In the third script (03_metaweb_comparisons.Rmd), we provide the statistical comparisons between our metaweb and other empirical metawebs as an R Markdown document (see Technical Validation: Comparison to other empirical metawebs), reproducing Fig. 4. We additionally provide a Python Jupyter Notebook document, outlining the error validation of the data extraction process (04_error_validation.ipynb and an accompanying.html file). Finally, we provide a script to reproduce Fig. 1 (05_metaweb_summary_figure_1.R).
